# Antibiotic resistance and virulence profile of *Klebsiella pneumoniae* isolated from wild Sumatran Orangutans (*Pongo abelii*)

**DOI:** 10.5455/javar.2024.k858

**Published:** 2024-12-29

**Authors:** Usamah Afiff, Rahmat Hidayat, Agustin Indrawati, Titiek Sunartatie, Aprilia Hardiati, Dordia Anindita Rotinsulu, Raden Iis Arifiantini, Deandarla Naoremisa, Nurhashunatil Mar’ah, Safika Safika

**Affiliations:** 1 Division of Medical Microbiology, School of Veterinary Medicine and Biomedical Sciences, IPB University, Bogor, Indonesia; 2 Division of Reproduction and Obstetrics, School of Veterinary Medicine and Biomedical Sciences, IPB University, Bogor, Indonesia; 3 Student of School of Veterinary Medicine and Biomedical Sciences, IPB University, Bogor, Indonesia; 4 Faculty of Vocation, Study Program of Veterinary Paramadics, Hasanuddin University, Makassar, Indonesia

**Keywords:** Antibiotic resistance, *K. pneumoniae*, multi-drug resistant, virulence factors, wild sumatran orangutans

## Abstract

**Objective:**

Orangutans (*Pongo abelii*), as endemic primates of Indonesia, are characterized by a predominantly arboreal lifestyle. *Klebsiella pneumoniae* (*K. pneumonia*) and other Gram-negative bacteria are present in the Indigenous flora of many mammals, including orangutans. This study aimed to investigate the antibiotic resistance and virulence profile of *K. pneumonia *isolated from wild Sumatran orangutans.

**Materials and Methods:**

This study investigated 10 fecal samples from wild Sumatran orangutans from the Gunung Leuser National Park, Aceh, Indonesia. Biochemical and molecular identification of *K. pneumoniae* using the RNA polymerase subunit b gene and detection of virulence-associated genes. In addition, molecular detection of antibiotic resistance genes was performed to characterize the resistance mechanisms in the isolates.

**Results:**

*K. pneumonia *was detected in 6 out of 10 fecal samples from wild Sumatran orangutans. The virulence genes *mrk*D and *ent*B were detected in all (100%) of the isolates, whereas wabG was identified in 83.33% of the strains. Antibiotic susceptibility testing against *K. pneumoniae* revealed that three isolates were susceptible to streptomycin (S) and nalidixic acid (NA), while all six isolates were susceptible to chloramphenicol and ciprofloxacin. One isolate demonstrated intermediate resistance to NA, while the remaining two exhibited intermediate resistance to S. Six isolates were resistant to ampicillin, tetracycline, and erythromycin, indicating multidrug resistance. Furthermore, antibiotic resistance genes were detected in the isolates with the following prevalence: *bla*_TEM_ gene (six isolates; 100%), *bla*_SHV_ (six isolates; 100%), *bla*_CTX-M _gene (four isolates; 66.67%), and *tet*A gene (four isolates; 66.67%).

**Conclusion:**

This study revealed the virulence and resistance profile of *K. pneumoniae* bacterium isolated from wild Sumatran orangutans, which is essential for formulating effective conservation and healthcare strategies.

## Introduction

Orangutans are members of the family Hominidae, which includes the giant apes and primates (*Pongo* spp.). They are one of the few extant giant ape species in Southeast Asia [1–3]. Orangutans have long limbs and reddish or brown hair. Only three species of orangutans are known to occur in Indonesia: *Pongo pygmaeus* (*P. pygmaeus*) on the island of Borneo, *Pongo abelii,* and *Pongo tapanuliensis *(*P. tapanuliensis*) on the island of Sumatra [4–6]. The International Union for the Conservation of Nature has listed all three orangutan species as critically endangered due to population declines [7,8]. Orangutans are predominantly arboreal, living in and around trees. In general, Sumatran orangutans generally engage in social, feeding, and resting daily activities. Sumatran orangutans relax by sleeping at night and taking midday naps between activities. Sumatran orangutans typically build their shelters in trees. Orangutans live in semi-solitary communities. Social behaviors commonly observed in orangutans include mother-child interactions and juvenile orangutans engaged in play [9]. Sumatran orangutans primarily feed during the morning and afternoon, engaging in foraging behavior. Orangutans sample and smell their food before consuming it, discarding items that are unpalatable and searching for alternative sources. Although primarily frugivorous, their diet also includes bark, seeds, leaves, vines, flowers, and bark. In addition, orangutans often consume insects for protein [10,11].

The presence of gram-negative bacteria, such as *Klebsiella pneumoniae (K. pneumoniae)*, in the normal flora of several mammals [12–14] underscores the possibility of cross-species transmission of infectious agents. Understanding the dynamics of bacterial transmission and antibiotic resistance in wildlife populations is critical, as evidenced by the impact of *K. pneumoniae *infections on immunocompromised and apparently healthy orangutans. Several virulence factors of *K. pneumoniae* enable this bacterium to evade the host’s innate immune mechanisms. Capsules, exopolysaccharides associated with mucoviscosity, lipopolysaccharides, adhesins, and iron uptake systems are virulence factors of *K. pneumoniae* [15–17]. Infection caused by *K. pneumoniae* is exacerbated by its ability to cause nosocomial infections and its resistance to multiple antibiotics [18,19]. The emergence of antibiotic resistance in *K. pneumoniae* is a global concern [20–22], and the resistance of this bacterium in orangutan populations suggests the need for careful monitoring of environmental contamination and potential sources of antibiotic exposure.

This study aimed to enlighten the antibiotic resistance and virulence profile of *K. pneumoniae* isolated from wild Sumatran orangutans. This research has significant implications for the conservation of endangered species, including orangutans. We can strengthen conservation efforts to better address environmental challenges by identifying *K. pneumoniae* and its antibiotic resistance profile. This, in turn, will facilitate the implementation of strategies that effectively prevent the spread of antibiotic resistance in wildlife populations. As such, this study serves as an important scientific resource and makes a valuable contribution to both animal conservation and the preservation of natural habitats in Indonesia.

## Materials and Methods

### Ethical approval

This study received approval and a written letter of recommendation from the Ministry of Environment and Forestry Directorate General of Natural Resources and Ecosystem Conservation (*Kementerian Linkugnan Hidup dan Kehutanan, Direktorat Jenderal Konservasi Sumber Daya Alam dan Ekosistem*) Number SK 433/KSDAE/SET.3/KSA.2/8/2021.

### Sample collection

Ten fecal samples were collected from adult Sumatran orangutans (*Pongo abelii*) living in Gunung Leuser National Park, Southeast Aceh Regency, Indonesia. The samples were collected from wild orangutans between 11 and 15 years old. No treatments were administered to these orangutans. Gunung Leuser National Park is located at 903°02’50.5” north latitude and 097°25’02.0” east longitude and covers an area of 281,574.62 hectares. Regarding topography, the regions range from coastal areas (above 0 meters above sea level [MASL]) to alpine areas (up to 3,000 MASL). About 80% of the topography has a more than 40% slope. In this jungle environment, orangutans thrive without human intervention for health management, nutrition, vaccination, or deworming. Their diet consists of fruit harvested and eaten directly from the trees. They live in nesting trees between 15 and 40 m high. The feces of the orangutans that fell from the trees were collected. To avoid environmental contamination, only the central part of the feces of recently defecated orangutans was collected. The samples were placed in plastic bags and transported in a cool box to the laboratory, where the temperature was kept between 2°C and 10°C [9].

### Biochemical methods for identification

Samples were cultured on MacConkey agar (MAC) differential selective medium CM0007 (Oxoid, UK) for 24 h at 37°C. Colonies of *K. pneumoniae* can be identified macroscopically by observing mucoid formation and the ability of the bacteria to ferment lactose. Single colonies were then subcultured on tryptic soy agar (TSA) CM0131 (Oxoid, UK) for purification. Bacterial colonies from the TSA culture were picked, placed on a glass plate, and stained with Gram’s stain for microscopic identification. The 3% potassium hydroxide test was also performed to confirm that the bacteria were Gram-negative. The oxidase test was then carried out by dropping some oxidase reagents. In addition, indole, methyl red, voges-proskauer, and Simmon citrate (indole MB0209, methyl red-Voges-Proskauer CM1092, and Simmon citrate CM0155B), triple sugar iron agar (TSIA) CM0277B, Christensen’s urea agar CM0053, and carbohydrate fermentation tests (Oxoid, UK) were performed for biochemical characterization. *K. pneumoniae* is positive for the methyl red test, Simmon’s citrate test, urease test, gas and sugar fermentation tests on TSIA, and the glucose, lactose, sucrose, maltose, dulcitol, and mannitol fermentation tests, but negative for the indole, Voges Proskauer, and hydrogen sulfide (H_2_S) tests [23].

### DNA extraction and molecular characterization

DNA extraction was performed using the Geneaid Presto™ Mini gDNA Bacteria Kit (Geneaid Biotech Ltd., New Taipei City, Taiwan) according to the manufacturer’s instructions. Identification of putative *K. pneumoniae* isolates based on biochemical characterization was confirmed by the detection of the RNA polymerase subunit b* (rpo*B) gene using polymerase chain reaction (PCR). The following primers were used to detect the *rpoB* gene: forward primer 5’-AAC CAG TTC CGC GTT GGC CTG A-3’ and reverse primer 5’-CCT GAA CAA CAC GCT CGG A-3’. The amplicon size was 1090 bp [24,25].

The PCR mix (25 μl/reaction) was composed as follows: 3 µl DNA template, 2 µl forward primers (10 M), 2 µl reverse primers (10 M), 12.5 µl of MytaqTM HS Red Mix (Bioline, UK), and 5.5 µl ddH_2_O. The PCR was carried out using a GeneAmp® PCR System 9700 thermal cycler (Applied BiosystemsTM, USA) with the following conditions: 4 min at 94°C (predenaturation), 30 sec of denaturation at 94°C, 1 min of annealing at 54°C, and 4 min of extension at 78°C, for 30 cycles. The final extension was carried out at 72°C for 5 min [24]. PCR results were detected by 1% agarose gel electrophoresis (Thermo Fisher Scientific USA).

### Detection of virulence-associated genes of K. pneumoniae

Virulence-associated genes of *K. pneumoniae* were identified using PCR. Primer sequences used to amplify fragments of the targeted virulence factor genes *rmp*A, *mag*A, *mrk*D, and *ent*B are shown in Table 1. The amplification conditions were as follows: initial denaturation at 94°C for 4 min, followed by 30 cycles of denaturation at 94°C for 30 sec, annealing at 54°C–59°C for 30 sec, and extension at 72°C for 1 min. A final extension was performed at 72°C for 10 min.

### Antibiotic susceptibility testing

The antibiotic susceptibility test was conducted using the Kirby-Bauer disc diffusion method. The turbidity level was compared to McFarland standards of 0.5 or 1.5 108 colony forming unit/ml. Suspensions were pipetted using a micropipette and dropped onto Mueller-Hinton agar (Oxoid, UK). Antibiotic discs of ampicillin (AMP; 10 μg), chloramphenicol (C; 30 μg), ciprofloxacin (CIP; 5 μg), nalidixic acid (NA; 30 μg), tetracycline (TET; 30 μg), erythromycin (E; 15 μg), and streptomycin (S; 10 μg) were used. The isolates were then incubated at 37°C for 18 h. Bacterial susceptibility was determined by measuring the size of the zone of inhibition and interpreting the results according to the Clinical and Laboratory Standards Institute’s 2023 guidelines [31].

**Table 1.** Primers to detect *rpoB,* virulence-associated genes, and antimicrobial resistance genes.

Gene target	Nucleotide sequence (5’-3’)	Amplicon (bp)	Reference
*rpoB*	F: 5'-ACCAGTTCCGCGTTGGCCTGG-3' R: 5'-CCTGAACAACACGCTCGGA-3'	1090	[25]
*magA*	F: 5'-GGTGCTCTTTACATCATTGC-3' R: 5'-GCAATGGCCATTTGCGTTAG-3'	1.283	[26]
*rmpA*	F: 5'-ACTGGGCTACCTCTGCTTCA-3' R: 5'-CTTGCATGAGCCATCTTTCA-3'	535	[26]
*mrkD*	F: 5'-CCACCAACTATTCCCTCGAA-3' R: 5'-ATGGAACCCACATCGACATT-3'	240	[26]
*entB*	F: 5'-ATTTCCTCAACTTCTGGGGC-3' R: 5'-AGCATCGGTGGCGGTGGTCA-3'	371	[26]
*wabG*	F: 5'-CGGACTGGCAGATCCATATC-3' R: 5'-ACCATCGGCCATTTGATAGA-3'	683	[27]
*blaTEM*	F: 5'-ATTTCCGTGTCGCCCTTAT-3' R: 5'-CTACGATACGGGAGGGCTTA-3'	516	[28]
*blaSHV*	F: 5'-CCTGTTAGCCACCCTGCC-3' R: 5'-CCGCAGATAAATCACCAC-3'	768	[28]
*blaCTX-M*	F: 5'-ATGATGAAAAAATCGTTATGC-3' R: 5'-CAGCATCTCCCAGCCTAAT-3'	866	[28]
*tetA*	F: 5'-GTA ATT CTG AGC ACT GTC GC-3' R: 5'-CTG CCT GGA CAA CAT TGC TT-3'	965	[28]
*qnrS*	F: 5'-ACG ACA TTC GTC AAC TGC AA-3' R: 5'-TAA ATT GGC ACC CTG TAG GC-3'	417	[29]
*ermB*	F: 5'-GAAAAGGTACTCAACCAAATA-3' R: 5'- GTAACGGTACTTAAATTGTTTAC-3'	639	[30]

### Detection of antibiotic resistance genes

Detection of the resistance genes *bla*_SHV_, *bla*_TEM, and_ *bla*_CTXM_ (AMP); *tet*A (TET); *qnr*S (NA and CIP); *erm*B (E); and *aac* (3)-IV (S) was performed only in *K. pneumoniae* isolates that showed phenotypic intermediate susceptibility and resistance. Antibiotic resistance genes were identified by PCR using the primers listed in Table 1.

## Results

### Bacterial isolation and identification

**Figure 1.**
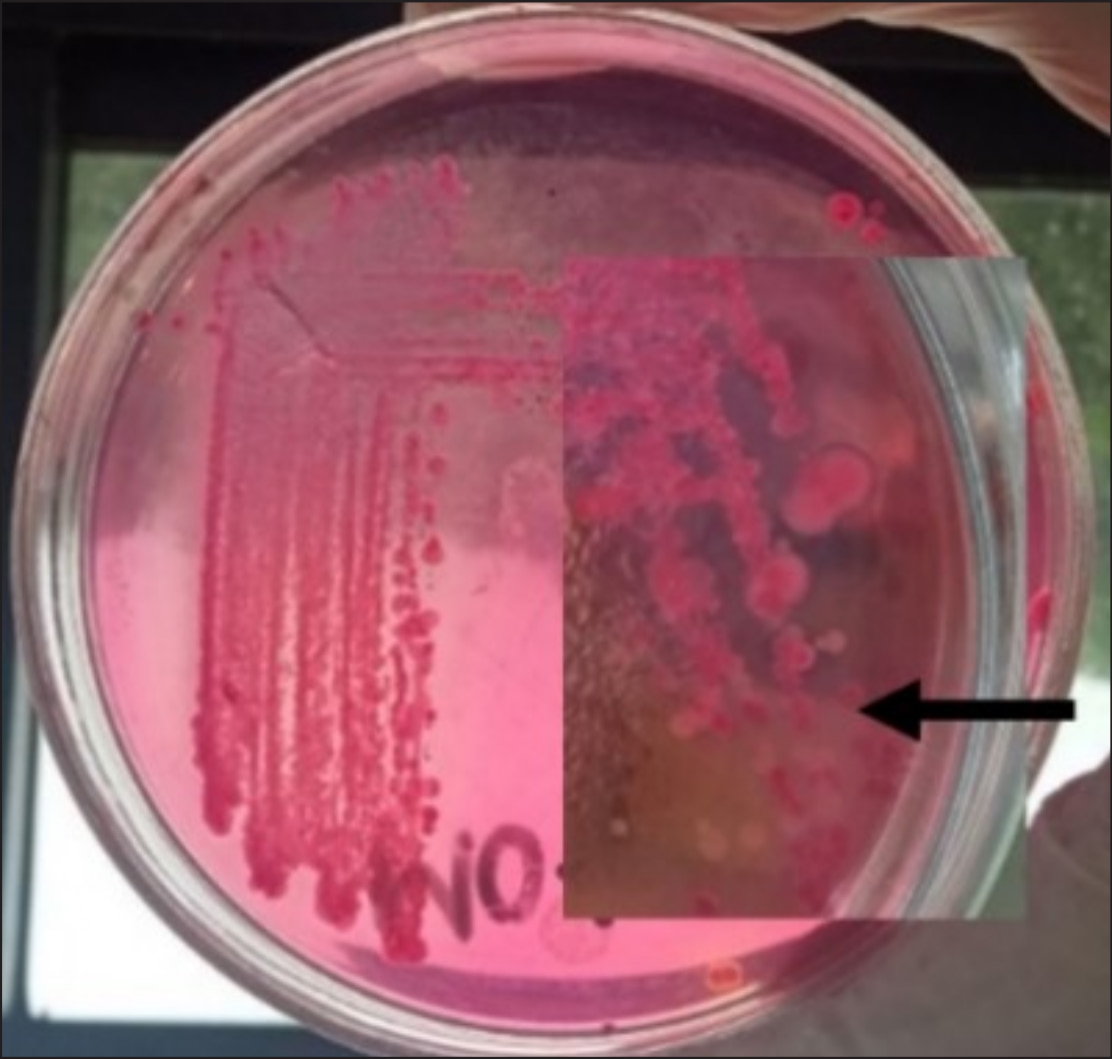
Isolation of *Klebsiella* bacteria from wild Sumatran orangutan samples. The morphology of *Klebsiella* genus colonies on MCA are convex, pink, and mucoid.

Six out of ten fecal samples (60%) from wild orangutans in Gunung Leuser National Park were positive for *Klebsiella* sp. Based on colony morphology and biochemical tests, we identified these isolates as *Klebsiella* spp. On MAC, the *Klebsiella* colonies appeared convex, mucoid, and fermented lactose, as indicated by a pink coloration (Fig. 1). The TSIA test characterized *Klebsiella* spp. as producing acid at the base and slope and did not produce gas or H_2_S. Positive results in the methyl red test, Simmons citrate test, and urease test were observed. The fermentation of glucose, sucrose, lactose, maltose, dulcitol, and mannitol occurs during the carbohydrate test. The resulting bacteria are indole and Voges-Proskauer negative and nonmotile.

**Figure 2.**
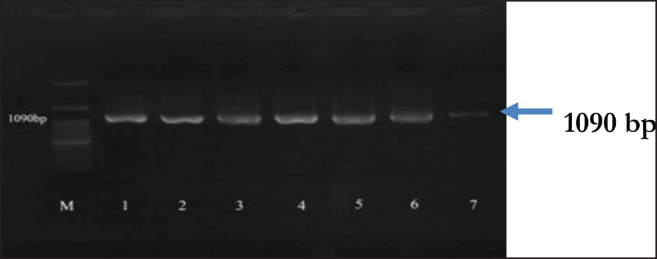
Amplification of the *rpo*B gene (1090 bp) isolates* K. pneumoniae *from wild Sumatran orangutans. M: 100 bp DNA marker; 2. ATCC: Positive control, 2–7: Positive isolates of *K. pneumoniae*.

Putative *K. pneumoniae* isolates, identified through biochemical characterization, were further confirmed by detecting the *rpo*B gene, which encodes the RNA polymerase subunit β. All isolates displayed a positive result for the *rpo*B gene. Positive isolates exhibited a band of 1090 bp, consistent with the results of the molecular identification (Fig. 2).

### Virulence-associated genes of K. pneumoniae

**Figure 3.**
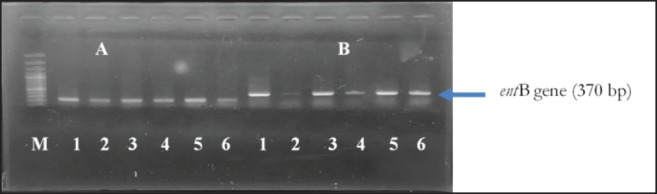
Amplification of the virulence factor *K. Pneumoniae* from wild Sumatran orangutans*. *(A) *mrk*D gene (240 bp); (B) *ent*B gene (370 bp). M: 100 bp DNA marker; 1–6: Positive isolates.

**Figure 4.**
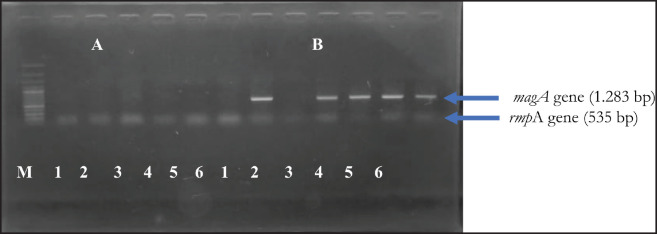
Amplification of the virulence factors of *K. Pneumoniae* from wild Sumatran orangutans. (A) *rmp*A gene (535 bp) and *magA *gene (1.283 bp). M: 100 bp DNA marker 1–6: Negative isolates; (B) *wabG* gene (683 bp). 1–6: Positive isolates.

This study investigated five virulence-associated genes, namely *rmp*A, *mag*A, *mrk*D, *ent*B, and *wab*G. All isolates detected the mrkD and entB genes, while five isolates (83.33%) detected the wabG gene. None of the isolates had the rmpA or magA genes detected. Figures 3 and 4 and Table 2 present these results.

### Antibiotic resistance genes of K. pneumoniae

The results of antibiotic susceptibility testing of *K. pneumoniae* isolates showed that all six isolates were susceptible to C and CIP, while three isolates were susceptible to S and NA. Two isolates showed intermediate susceptibility to S, and one isolate showed intermediate susceptibility to NA. All isolates were resistant to AMP, TET, and E; two isolates were resistant to NA, and one isolate was resistant to S (Table 3).

All isolates in this study exhibited resistance to three or more classes of antibiotics, a characteristic commonly defined as multidrug resistance (MDR). MDR is the resistance of bacteria to three or more groups of antibiotics [32]. In this study, all isolates were MDR. Table 3 shows that there were four isolates: OU-1, OU-3. OU-5 and OU-7 were resistant to three types of antibiotics, one isolate (OU-9) was resistant to four antibiotics, while another isolate (OU-10) was resistant to five types of antibiotics.

**Figure 5.**
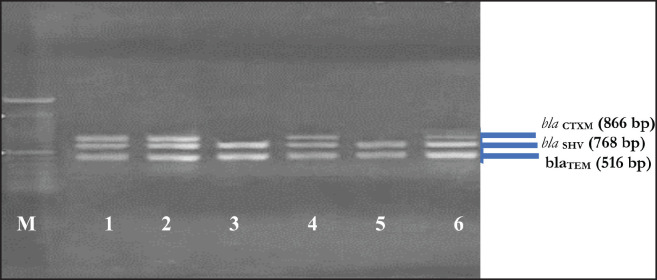
Amplification resistance genes of the *bla*_TEM_ (516 bp), *bla*_SHV_ (768 bp) and *bla*_CTXM_ (866 bp). M: 100 bp DNA marker, 1–6:* K. pneumoniae *isolates from wild Sumatran orangutans.

**Figure 6.**
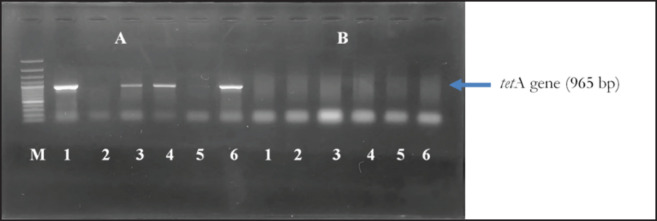
Amplification resistance genes of *K. pneumonia *from wild Sumatran orangutans M: 100 bp DNA marker, (A) *tet*A gene (965 bp) 1, 3, 4 and 6 Positive isolates; (B) *ermB *gene (639 bp) 1–6: negative isolates.

Phenotypically resistant* K. pneumoniae *isolates were subsequently tested for resistance genes. The following resistance genes were detected*: blaTEM* (6 isolates; 100%), *bla*_SHV_ (6 isolates; 100%), *bla*_CTX-M _gene (4 isolates; 66.67%), *tet*A gene (4 isolates; 66.67%), *qnr*S gene (0 isolates; 0%), *erm*B gene (0 isolates; 0%), and aac(3)-IV (0 isolates; 0%). The results are shown in Figures 5 and 6 and Table 4.

## Discussion

*K. pneumoniae* has been identified in the gastrointestinal tract of a wide range of animal species, including orangutans [33,34]. *K. pneumoniae*, a common gut microbiota member, has the potential to become pathogenic and cause infections, especially in immunocompromised individuals [35,36]. Although data on *K. pneumoniae* infections in Sumatran orangutans are limited, it is crucial to highlight that infectious diseases, including those caused by *K. pneumoniae*, pose significant threats to the survival and well-being of these endangered animals. *K. pneumoniae* infections can potentially cause various diseases in orangutans, including pneumonia, bacteremia, and additional systemic infections. The lack of access to medical intervention and treatment for these infections is particularly problematic in wild orangutan populations. Factors such as human-animal interactions, habitat degradation, and environmental changes can increase stress levels and susceptibility to infections in wildlife, particularly endangered species like the Sumatran orangutan.

**Table 2.** Virulence factors profile of *K. pneumoniae *isolated from wild Sumatran orangutans.

Isolate	*rmpA*	*magA*	*mrkD*	*entB*	*wabG*
OU-1	−	−	+	+	+
OU-3	−	−	+	+	-
OU-5	−	−	+	+	+
OU-7	−	−	+	+	+
OU-9	−	−	+	+	+
OU-10	−	−	+	+	+

Description: (+) = positive result, (−) = negative result for the gene which being tested.

**Table 3.** Antimicrobial resistance patterns of *K. pneumoniae *isolated from wild Sumatran orangutans.

	Antimicrobial susceptibility for each sample					
Antibiotics	OU-1	OU-3	OU-5	OU-7	OU-9	OU-10
AMP	R	R	R	R	R	R
TET	R	R	R	R	R	R
CIP	S	S	S	S	S	S
NA	I	S	S	S	R	R
C	S	S	S	S	S	S
E	R	R	R	R	R	R
Streptomycin	I	I	S	S	S	R
MDR	Yes	Yes	Yes	Yes	Yes	Yes

Description: I = intermediet, R = resistant, S = sensitive.

**Table 4.** Profile resistance genes of *K. pneumonia *isolated from wild Sumatran orangutans.

*Isolate*	*bla_TEM_*	*bla_SHV_*	*bla_CTX-M_*	*tetA*	*qnrS*	*ermB*	*aac (3)-IV*
OU-1	+	+	+	+	X	−	X
OU-3	+	+	+	−	X	−	X
OU-5	+	+	−	+	X	−	X
OU-7	+	+	+	+	X	−	X
OU-9	+	+	−	−	−	−	X
OU-10	+	+	+	+	−	−	−

Description: (+) = positive result, (−): negative resultfor the gene which being tested, X: test not performed.

The presence of *K. pneumoniae *isolates from wild Sumatran orangutans, all carrying the *mrk*D and *ent*B genes, indicates a common genetic trait among these strains. These genes play crucial roles in the pathogenicity and virulence of *K. pneumoniae* [17,19,37]. The *mrk*D gene, which is associated with the synthesis of type 3 fimbriae, enhances the adherence of the bacterium to host cells, thereby facilitating the processes of colonization and eventual infection [38]. By enhancing the ability of the bacterium to colonize and infect the host, this adhesion mechanism contributes to its pathogenesis. The detection of the *mrk*D gene in all strains suggests a possible common virulence mechanism among the strains of *K. pneumoniae* in the orangutan population.

Similarly, the *ent*B gene is involved in iron acquisition via enterobactin, a siderophore. Iron is a critical nutrient for bacterial growth, and the ability of *K. pneumoniae* to scavenge iron from the host environment enhances its survival and development [39–41]. The presence of the *ent*B gene in each isolate highlights the critical role of this mechanism in the pathogenicity and persistence of *K. pneumoniae* in the orangutan gastrointestinal tract.

The presence of the *wab*G gene in 83.33% of the isolates suggests potential resistance to fosfomycin, a commonly prescribed antibiotic [42]. This finding emphasizes the importance of understanding the antimicrobial resistance profile of these bacterial strains and the need for comprehensive antimicrobial stewardship initiatives to prevent the spread of resistance within wildlife populations. The absence of the *rmp*A and *mag*A genes in all isolates indicates a unique genetic feature distinguishing these strains from certain strains of hypermucoviscous *K. pneumoniae* associated with severe human infections [43,44]. The absence of these genes in the *K. pneumoniae* strains from the Sumatran orangutan population suggests that they have distinct virulence potential. The results of this study improve the understanding of the genetic characteristics and virulence components of *K. pneumoniae* strains that infect orangutans. As a result, there is now a clearer understanding of the precise mechanisms by which these strains can induce infection and promote pathogenesis.

Antibiotics are frequently derived from natural sources, including specific microorganisms that generate them for defense. For example, E is produced by *Streptomyces erythraeus* [45,46]. Beta-lactam and TET can also be synthesized by environmental microorganisms. TETs are formed through the fermentation of *actinomycetes*, and the antibiotic penicillin is naturally produced by *Penicillium* mold [47,48]. Orangutans may encounter trace amounts of these antibiotic compounds by consuming vegetation, water, or other environmental components harboring antibiotic-producing microorganisms. This exposure has the potential to influence the gut microbiota of orangutans, potentially leading to the emergence of antibiotic resistance within their microbial communities.

Antimicrobial resistance is a global issue affecting various species, including wildlife [49,50]. In orangutans, resistance can arise from various factors, including ingestion of antibiotic compounds present in the environment, overuse and inappropriate use of antibiotics in veterinary and human medicine, and environmental contamination. Human-wildlife interactions potentially also introduce antibiotic-resistant microorganisms. The emergence and spread of antibiotic-resistant bacteria are often linked to inappropriate and excessive use of antibiotics in veterinary medicine and human and animal populations. In addition, environmental factors such as contamination and the release of antibiotics into the environment can contribute to the development of antibiotic resistance in various bacterial strains.

All isolates in this study exhibited resistance to three or more classes of antibiotics, a characteristic commonly defined as MDR. The discovery of MDR isolates in this study underscores the urgent concern about antimicrobial resistance in *K. pneumoniae* infections in orangutans. MDR strains are a major concern because they are resistant to a wide range of antibiotics, making the treatment of infections caused by these strains much more difficult [51,52]. Particularly for endangered species like orangutans, the presence of MDR isolates in this study highlights the importance of understanding the mechanisms underlying antibiotic resistance in wildlife populations. Comprehensive surveillance programs and strategic interventions are urgently needed to manage and reduce the spread of MDR strains in the environment.

A comprehensive strategy would be required to reduce the risk of antimicrobial resistance and MDR *K. pneumoniae* in orangutans. This would include advocating the responsible use of antibiotics in animal and human healthcare, establishing efficient waste management systems, and limiting human-wildlife interactions that can facilitate the spread of resistant bacteria. Understanding and addressing the potential impact of antimicrobial resistance on endangered species, such as the Sumatran orangutan, requires vigilant monitoring of wildlife health. Understanding the prevalence and characteristics of MDR isolates in orangutans is critical not only for health management but also for advancing conservation initiatives aimed at protecting these endangered species. This information can serve as a basis for informed decision-making and the formulation of targeted interventions to protect the survival and welfare of orangutans.

As the sample size of wild orangutans in the study was limited, it is important to interpret these findings cautiously and consider the broader context of orangutan populations in their natural habitat. Observations of wild orangutans in various forest habitats reveal diverse behaviors, such as arboreal activity and wide-ranging movement in the forest. The dispersed population of around 30 wild orangutans across hundreds of hectares of forest illustrates the difficulties of conducting in-depth research and monitoring in large and isolated habitats. The challenges include sample collection limitations, operational constraints, and the complications associated with studying animals in their natural, often inaccessible, habitats. Despite these limitations, this study contributes significantly to understanding the prevalence and genetic characteristics of *K. pneumoniae* within the sampled orangutan population. To assess the broader prevalence and distribution of this bacterium among orangutans in different forest areas and their native habitats, further research is necessary.

Further research and surveillance initiatives should include the potential use of non-invasive sampling methods and advanced genetic analysis. To reduce the likelihood of *K. pneumoniae* infection in wildlife populations, including Sumatran orangutans, it is imperative to prioritize habitat conservation and protection, establish disease surveillance initiatives, and advocate for responsible tourism and human-wildlife interaction protocols. A comprehensive understanding of the prevalence, transmission dynamics, virulence factors, and resistance of *K. pneumoniae* in orangutans is essential to formulating effective health and conservation strategies. Enforcement of environmental management practices, responsible antibiotic use, and comprehensive surveillance are imperative to prevent the spread of infection and the emergence of antibiotic resistance in orangutans and other wildlife populations. To ensure the conservation and welfare of Sumatran orangutans and their ecosystems, conservation organizations, local communities, researchers, and government agencies must work together to improve our understanding of disease dynamics and health status.

## Conclusion

*K. pneumoniae* was found in the feces of wild Sumatran orangutans. The virulence of *K. pneumoniae* was determined by the presence of the *mrk*D, *ent*B, and *wab*G genes. *K. pneumoniae* isolates obtained from wild Sumatran orangutans exhibited resistance to multiple antibiotics, including AMP, TET, and E, indicating MDR. Understanding the prevalence, virulence factors, and resistance of *K. pneumoniae* in orangutans is essential for developing effective conservation and healthcare strategies.

## List of abbreviations

AMP, ampicillin; C, chloramphenicol; CIP, ciprofloxacin; E, erythromycin; H_2_S, hydrogen sulfide; MAC, MacConkey agar; MASL, meters above sea level; MDR, multidrugs resistant; MHA, Mueller-Hinton Agar; NA, nalidixic acid; PCR, polymerase chain reaction; *rpoB*, RNA polymerase subunit b; S, streptomycin; TET, tetracycline; TSA, tryptic soy agar; TSIA, triple sugar iron agar.

## References

[1] Habinger SG, Chavasseau O, Jaeger JJ, Chaimanee Y, Soe AN, Sein C (2022). Evolutionary ecology of Miocene hominoid primates in Southeast Asia. Sci Rep.

[2] Santika T, Sherman J, Voigt M (2022). Effectiveness of 20 years of conservation investments in protecting orangutans. Curr Biol.

[3] Spehar SN, Sheil D, Harrison T, Louys J, Ancrenaz M, Marshall AJ (2018). Orangutans venture out of the rainforest and into the Anthropocene. Sci Adv.

[4] Nater A, Mattle-Greminger MP, Nurcahyo A, Nowak MG, de Manuel M, Desai T (2017). Morphometric behavioral, and genomic evidence for a new orangután species. Curr Biol.

[5] Meijaard E, Ni’matulalah S, Dennis R, Sherman J, Wich SA (2021). The historical range and drivers of decline of Tapanuli Orang utan. PLoS One.

[6] Safika S, Wardinal W, Ismail YS, Nisa K, Sari WN (2019). *Weissella*, a novel lactic acid bacteria isolated from wild Sumatran orangutans (*Pongo abelii*). Vet world.

[7] IUCN (2021). Conflict and conservation. Nature in a globalised world report No.1. Gland. IUCN, Switzerland, Europe.

[8] KSDAE (2016). Directorate general of natural resources and ecosystem conservation. https://www.ksdae.menlhk.go.id.

[9] Safika S, Indrawati A, Afiff U, Hastuti YT, Zureni Z, Jati AP (2023). First study on profiling of gut microbiome in wild and captive Sumatran orangutans (*Pongo abelii*). Vet World.

[10] Zahro YRD, Mardiastuti A, Rahman DE (2021). Behavior and food of reintroduced Bornean orangutan (*Pongo pygmaeus wurmbii*) at feeding site and forest area in Lamandau wildlife sanctuary. In Proceedings of the 7th International Conference on Biological Science (ICBS.

[11] Safika S, Indrawati A, Afif U, Hidayat R, Sunartatie T (2023). Metagenomic analysis of mycobiome in wild and captivity Sumatran orangutans (*Pongo abelii*). J Adv Vet Anim Res.

[12] Martin RM, Bachman MA (2018). Colonization, infection, and the accessory genome of *Klebsiella pneumoniae*. Front Cell Infect Microbiol.

[13] Chen Q, Wang M, Han M, Xu L, Zhang H (2023). Molecular basis of *Klebsiella pneumoniae* colonization in host. Microb Pathog.

[14] Nakhaee P, Zarif Moghadam H, Shokrpoor S, Razmyar J (2022). *Klebsiella pneumoniae* infection in canaries (*Serinuscanaria domestica*): a case report. Iranian J Vet Res.

[15] Zhang Z, Lei L, Zhang H, Dai H, Song Y, Li L (2021). Molecular investigation of *Klebsiella pneumoniae* from clinical companion animals in Beijing, China, 2017–2019. Pathogens.

[16] Davies YM, Cunha MP, Oliveira MG, Oliveira MC, Philadelpho N, Romero DC (2016). Virulence and antimicrobial resistance of *Klebsiella pneumoniae* isolated from passerine and psittacine birds. Avian Pathol.

[17] Riwu KHP, Effendi MH, Rantam FA, Khairullah AR, Widodo A (2022). A review: virulence factors of *Klebsiella pneumoniae* as emerging infection on the food chain. Vet World.

[18] Vuotto C, Longo F, Balice MP, Donelli G, Varaldo PE (2014). Antibiotic resistance related to biofilm formation in *Klebsiella pneumoniae*. Pathogens.

[19] Lee CR, Lee JH, Park KS, Jeon JH, Kim YB, Cha CJ (2017). Antimicrobial resistance of hypervirulent *Klebsiella pneumoniae*: epidemiology, hypervirulence-associated determinants, and resistance mechanisms. Front Cell Infect Microbiol.

[20] Choby JE, Howard-Anderson J, Weiss DS (2020). Hypervirulent *Klebsiella pneumoniae*—clinical and molecular perspectives. J Intern Med.

[21] Mancuso G, Midiri A, Gerace E, Biondo C (2021). Bacterial antibiotic resistance: the most critical pathogens. Pathogens.

[22] Pulingam T, Parumasivam T, Gazzali AM, Sulaiman AM, Chee JY, Lakshmanan M (2022). Antimicrobial resistance: prevalence, economic burden, mechanisms of resistance and strategies to overcome. Eur J Pharm Sci.

[23] Safika S, Nilasari Z, Pasaribu FH (2022). Detection of antibiotic resistance coding gene in *Klebsiella pneumoniae* bacteria isolated from broiler chickens in West Java, Indonesia. J Appl Pharm Sci.

[24] Alves MS, Dias RC, de Castro AC, Riley LW, Moreira BM (2006). Identification of clinical isolates of indole-positive and indole-negative *Klebsiella* spp. J Clin Microbiol.

[25] El Fertas-Aissani R, Messai Y, Alouache S, Bakour R (2013). Virulence profiles and antibiotic susceptibility patterns of *Klebsiella pneumoniae* strains isolated from different clinical specimens. Pathol Biol.

[26] Brisse S, Fevre C, Passet V, Issenhuth-Jeanjean S, Tournebize R, Diancourt L (2009). Virulent clones of *Klebsiella pneumoniae*: identification and evolutionary scenario based on genomic and phenotypic characterization. PLoS One.

[27] Colom K, Pérez J, Alonso R, Fernández-Aranguiz A, Lariño E, Cisterna R (2003). Simple and reliable multiplex PCR assay for detection of blaTEM, bla(SHV) and blaOXA-1 genes in *Enterobacteriaceae*. FEMS Microbiol Lett.

[28] Chuah LO, Shamila Syuhada AK, Mohamad Suhaimi I, Farah Hanim T, Rusul G (2018). Genetic relatedness, antimicrobial resistance and biofilm formation of *Salmonella* isolated from naturally contaminated poultry and their processing environment in northern Malaysia. Food Res Int.

[29] Robicsek A, Strahilevitz J, Sahm DF, Jacoby GA, Hooper DC (2006). qnr prevalence in ceftazidime-resistant *Enterobacteriaceae* isolates from the United States. Antimicrob Agents Chemother.

[30] Song JH, Chang HH, Suh JY (2004). ANSORP Study Group. Macrolide resistance and genotypic characterization of *Streptococcus pneumoniae* in Asian countries: a study of the Asian network for surveillance of resistant pathogens (ANSORP). J Antimicrob Chemother.

[31] Clinical and Laboratory Standards Institute (CLSI) (2023). M100 performance standards for antimicrobial, Clinical and Laboratory Standards Institute, Wayne, PA.

[32] Magiorakos AP, Srinivasan A, Carey RB (2012). Multidrug-resistant, extensively drug-resistant and pandrug-resistant bacteria: an international expert proposal for interim standard definitions for acquired resistance. Clin Microbiol Infect.

[33] Gozalo AS, Elkins WR, Lambert LE, Stock F, Thomas ML 3rd, Woodward RA (2016). Genetic diversity of *Klebsiella pneumoniae* isolates during an outbreak in a non-human primate research colony. J Med Primatol.

[34] Anzai EK, de Souza Júnior JC, Peruchi AR, Fonseca JM, Gumpl EK, Pignatari ACC (2017). First case report of non-human primates (*Alouatta clamitans*) with the hypervirulent *Klebsiella pneumoniae* serotype K1 strain ST 23: a possible emerging wildlife pathogen. J Med Primatol.

[35] Liang Z, Wang Y, Lai Y, Zhang J, Yin L, Yu X (2022). Host defense against the infection of *Klebsiella pneumoniae*: new strategy to kill the bacterium in the era of antibiotics?. Front Cell Infect Microbiol.

[36] Fliss M, van den Berg CHSB, Kuijper E, Notermans DW, Hendrickx APA, Schoots MH (2022). Brief report: community-acquired Friedlander’s pneumonia and pulmonary metastatic *Klebsiella pneumoniae* infection caused by hypervirulent ST23 in the Netherlands. Eur J Clin Microbiol Infect Dis.

[37] Piazza A, Perini M, Mauri C, Comandatore F, Meroni E, Luzzaro F, Principe L (2022). Antimicrobial susceptibility, virulence, and genomic features of a hypervirulent serotype K2, ST65 *Klebsiella pneumoniae* causing meningitis in Italy. Antibiotics.

[38] Gomes AÉI, Pacheco T, Dos Santos CDS, Pereira JA, Ribeiro ML, Darrieux M (2021). Functional insights from KpfR, a new transcriptional regulator of fimbrial expression that is crucial for *Klebsiella pneumoniae *pathogenicity. Front Microbiol.

[39] Miethke M, Marahiel MA (2007). Siderophore-based iron acquisition and pathogen control. Microbiol Mol Biol Rev.

[40] Holden VI, Wright MS, Houle S, Collingwood A, Dozois CM, Adams MD (2018). Iron acquisition and siderophore release by carbapenem-resistant sequence type 258* Klebsiella pneumoniae*. mSphere.

[41] Ballén V, Gabasa Y, Ratia C, Ortega R, Tejero M, Soto S (2021). Antibiotic resistance and virulence profiles of *Klebsiella pneumoniae* strains isolated from different clinical sources. Front Cell Infect Microbiol.

[42] Fatima S, Liaqat F, Akbar A, Sahfee M, Samad A, Anwar M (2021). Virulent and multidrug-resistant *Klebsiella pneumoniae* from clinical samples in Balochistan. Int Wound J.

[43] Paczosa MK, Mecsas J (2016). *Klebsiella pneumoniae*: going on the offense with a strong defense. Microbiol Mol Biol Rev.

[44] Kikuchi S, Kosai K, Ota K, Mitsumoto-Kaseida F, Sakamoto K, Hasegawa H (2023). Clinical and microbiological characteristics of bloodstream infection caused by *Klebsiella pneumoniae* harboring rmpA in Japanese adults. Sci Rep.

[45] Wu J, Zhang Q, Deng W, Qian J, Zhang S, Liu W (2011). Toward improvement of erythromycin A production in an industrial *Saccharopolyspora erythraea* strain via facilitation of genetic manipulation with an artificial attB site for specific recombination. Appl Environ Microbiol.

[46] Chen D, Feng J, Huang L, Zhang Q, Wu J, Zhu X (2014). Identification and characterization of a new erythromycin biosynthetic gene cluster in actinopolysporaerythraea YIM90600, a novel erythronolide-producing halophilic actinomycete isolated from salt field. PLoS One.

[47] De Simeis D, Serra S (2021). Actinomycetes: a never-ending source of bioactive compounds-an overview on antibiotics production. Antibiotics.

[48] García-Estrada C, Martín JF, Cueto L, Barreiro C (2020). Omics approaches applied to *Penicillium**chrysogenum* and penicillin production: revealing the secrets of improved productivity. Genes.

[49] Bhowmik P, Modi B, Roy P, Chowdhury A (2023). Strategies to combat gram-negative bacterial resistance to conventional antibacterial drugs: a review. Osong Public Health Res Perspect.

[50] Antimicrobial Resistance Collaborators (2022). Global burden of bacterial antimicrobial resistance in 2019: a systematic analysis. Lancet.

[51] De Oliveira DMP, Forde BM, Kidd TJ, Harris PNA, Schembri MA, Beatson SA (2020). Antimicrobial resistance in ESKAPE pathogens. Clin Microbiol Rev.

[52] Russo A, Fusco P, Morrone HL, Trecarichi EM, Torti C (2023). New advances in management and treatment of multidrug-resistant *Klebsiella pneumoniae*. Expert Rev Anti Infect Ther.

